# The influence of depression on patient-reported outcomes for hip-fracture patients 1 year after surgery: a prospective cohort study

**DOI:** 10.1007/s40520-019-01207-5

**Published:** 2019-04-26

**Authors:** Paula Kelly-Pettersson, Bodil Samuelsson, Maria Unbeck, Olav Muren, Martin Magnéli, Max Gordon, André Stark, Olof Sköldenberg

**Affiliations:** 1 Department of Clinical Sciences at Danderyd Hospital, Karolinska Institutet, Stockholm, Sweden; 2 Department of Molecular Medicine and Surgery, Karolinska University Hospital, Karolinska Institutet, Stockholm, Sweden

**Keywords:** Hip fracture, Depression, Patient-reported outcomes, Hip function, Health-related quality of life

## Abstract

**Background:**

Depression is common in elderly hip-fracture patients and together with cognitive impairment is associated with increased risk of mortality.

**Aim:**

We aimed to examine the influence depression has on patient-reported outcome up to 1 year after acute hip fracture.

**Methods:**

162 hip-fracture patients participated in the prospective observational cohort study and were followed up at baseline, and 3 and 12 months using patient-reported outcome scores. Patients with cognitive impairment were excluded. Depression was defined as a score ≥ 8 on the depression subscale of the Hospital Anxiety Depression Scale (HADS D), having a diagnosis of depression or being treated with anti-depressant medication. Hip function was assessed using Harris Hip Score (HHS), EQ-5D was used to assess health status and Quality of life, and the Pain Numerical Rating Scale (PRNS) was used to assess pain levels. A linear regression model adjusted for group, age, sex, and ASA class was used to identify risk factors for functional outcome 12 months after fracture.

**Results:**

35 patients were included in the depression group versus 127 in the control group. No statistical differences were found in the demographic data (age, sex, ASA class, fracture type, operation method, living situation, activities of daily living ADL and clinical pathway) between the groups. In the regression model, we found no correlation between depression and the patient-reported outcome.

**Conclusion:**

In young elderly hip fracture patients without cognitive dysfunction, depression may not be of major importance for the rehabilitation of hip function in the short term.

## Introduction

In elderly patients with hip-fracture, depression is common [1] and the prevalence among this patient group has been reported to range from between 9 and 47% [2]. Depression has been associated with an increased risk of mortality in hip-fracture patients [3, 4], and coupled with impaired cognitive status has been found to be a significant predictor of poorer outcomes in recovery for these patients [5, 6]. In addition, one out of every five individuals who are not depressed at the time of their hip fracture is likely to develop depressive symptoms after 8 weeks [7].

Although some studies have shown that the presence of depression or depressive symptoms can adversely affect functional outcome after hip-fracture [5, 8–13], other studies have not found this association [14–17]; so the evidence is conflicting and further investigation is warranted.

## Objectives

The aim of this study was to examine the influence depression has on patient-reported outcome up to 1 year after hip fracture surgery.

## Patients and methods

### Study design and setting

This prospective observational cohort study was conducted at the Orthopaedic Department of Danderyd hospital between 2010 and 2013 (inclusion period 2010–2012). The hospital is one of the large acute care hospitals in the Stockholm metropolitan area, with an uptake area of approximately 500,000 inhabitants. The department has a 52-bed unit, where both elective and acute patients are treated, and around 650 patients with hip fractures are admitted each year. The study is reported in accordance with the STROBE guidelines [18].

### Participants

All patients presenting with an acute hip fracture with intact cognitive function, a Short Portable Mental Status Questionnaire (SPMSQ) [19] score ≥ 7, who were independent walkers pre-fracture, and willing and able to participate in the study were eligible for inclusion. We excluded patients with pathological fractures, substance abuse, those unable to speak or understand Swedish, patients with multiple comorbidities whose condition was unstable post-operatively, patients belonging to other hospital health service districts as well as those patients considered unsuitable for any other reason, for example, profound deafness or blindness.

### Sample size

Inclusion in the study continued over a period of 2 years and this was considered a sufficient length of time to include a suitable sample size. No formal power calculation was carried out.

### Data collection

The administrative data which were collected from both the electronic patient records and the hospital administrative data collection systems included age, gender, type of fracture, operation method, the American Society of Anaesthesiologists’ physical status classification system, the ASA-score [20], the clinical management pathway, and Ambulance versus Hip process pathway. The Ambulance pathway was a newly introduced admission system to fast-track hip-fracture patients directly to X-ray and then the orthopaedic ward bypassing the Accident Emergency Department and the Hip process was the normal admission pathway. Which pathway the patient was admitted via was dependant on which ambulance provider transported the patient to hospital. Data were also collected regarding the length of hospital stay (in days), waiting time to surgery in h:m and date of death.

The patients were followed up at 3 and 12 months after their fracture with a visit to a research nurse (RN) (PKP) or by a telephone interview. Data were also collected regarding the patients’ living situation pre-fracture, KATZ ADL score [21] and the Charnley classification of walking ability [22]. Patients gave details about their medical history and a review of the medical records was carried out by the research RN to identify those patients with a history of depression and/or use of anti-depressant medications. The occurrence of adverse events (AEs) was identified via patient-reported outcomes. The patients were asked by the research RN to verify or deny AEs from a pre-defined list. Four questionnaires were sent by post to the patients and were returned either by post or at the visit to the research RN or the information was collected during the telephone interview.

A digital case report form was completed for each patient and the data were collected and managed using the Research Electronic Data Capture (REDCap) electronic capture system [23] hosted by the Karolinska Institute. This is a secure, web-based application designed to support data capture for research studies.

### Outcomes

The functional outcome variables of interest were the disease-specific (modified) Harris Hip Score (HHS); the pain numerical rating scale (PRNS), the generic quality of life score EQ-5D-3L and the Hospital Anxiety and Depression Score (HADS). All were measured at baseline, and 3 and 12 months.

### Hip function

Hip function was assessed using a modified version of the Harris Hip Score (HHS). The score was initially developed for the evaluation of hip function for patients undergoing arthroplasty for traumatic arthritis after luxation or acetabular fractures [24]. In the original format, the assessment was carried out by the orthopaedic surgeon, but the instrument has since evolved into a self-reporting score for patients [25]. It has been validated for use in the hip-fracture patients with neck of femur fractures [25, 26] and generates a score of 0–100 points where a high score indicates a better hip function.

### Pain assessment

The Pain Numerical Rating Scale (PRNS) [27] was used to assess levels of pain. It is a 11-point numeric self-reporting scale ranging from 0 (no pain) to 10 (worst pain imaginable). The instrument is used widely, and is quick and easily used by patients.

### Quality of life

The EuroQol 5-dimension (EQ-5D) questionnaire is a generic instrument developed by the EuroQol group [28, 29], which is used to measure perceived quality of life and health status. It consists of five domains: pain, self-care, mobility, anxiety and depression, as well as, a visual analogue scale (EQ-VAS), which registers the patient’s perceived current health status on a scale from 0 to 100, where 0 indicates the worst possible health status and 100 the best possible or optimal status.

### The Hospital Anxiety and Depression Scale (HADS)

The Hospital Anxiety and Depression Scale (HADS) [30] is a self-evaluating instrument consisting of a 14-item questionnaire that was initially used as a screening tool to assess and detect levels of anxiety and depression in the primary health care setting. The score was not intended as a tool for clinical diagnosis [31], as other clinical symptoms need to be assessed before a diagnosis can be made.

It consists of two subscales, each with seven questions relating to depressive symptoms (HADS D) and seven questions relating to anxiety (HADS A). Each question gives a score from 0 to 3, thus the subtotals for each of the subscales, anxiety and depression can generate a score of between 0 and 21 points. The cut-off limits [32] for the scoring of the subscales of anxiety and depression are categorized as follows:

**Table:** 

0–7 points	Within normal range indicating no depression/anxiety
8–10 points	Mild depression/anxiety
11–14 points	Moderate severe depression/anxiety
15–21 points	Severe depression/anxiety

The questionnaire is easily administered and takes around 2–5 min to complete. The results of subscales can be used independently and in this study only the results of HADS D were used. The 1-year mortality rate and the occurrence of patient-reported AEs and serious adverse (SAEs) during the study are also presented.

### Exposure

The patients were divided into two groups, those with depression (depression group) at the start (baseline) of the study and those without (control group). Depression was defined as patients with the Hospital Anxiety and Depression Scale (HADS), depression subscale (HADS D) score of ≥ 8 points and/or a diagnosis of depression irrespective of current use of anti-depressive medications at the time of inclusion. This definition was chosen, as depressive illness, regardless of current symptoms, has a bearing on how individuals with musculoskeletal conditions experience symptoms [33, 34].

### Statistical methods

The variables were normally distributed and are presented as the mean with standard deviation and the mean difference is presented with 95% confidence interval. *p* values were derived from Student’s *T* test. A linear regression model was constructed to identify and adjust for factors, which apart from the exposure variable could influence the patient-reported outcome measures at 1 year. We adjusted for confounders with age, sex and ASA-classification as a proxy for pre-existing illness or co-morbidity and pre-fracture hip function, i.e. HHS, EQ-5D and PRNS. For the mortality and AE outcomes we used the chi-square test. The statistical analysis was performed using SPSS software for Macintosh (SPSS, Chicago, Illinois), version 25.0.

### Ethical considerations

All patients received both oral and written information about the study and gave their written informed consent to participate in the study. The study received approval from the Regional Ethics Committee in Stockholm.

## Results

### Participants and descriptive data

**Fig. 1:**
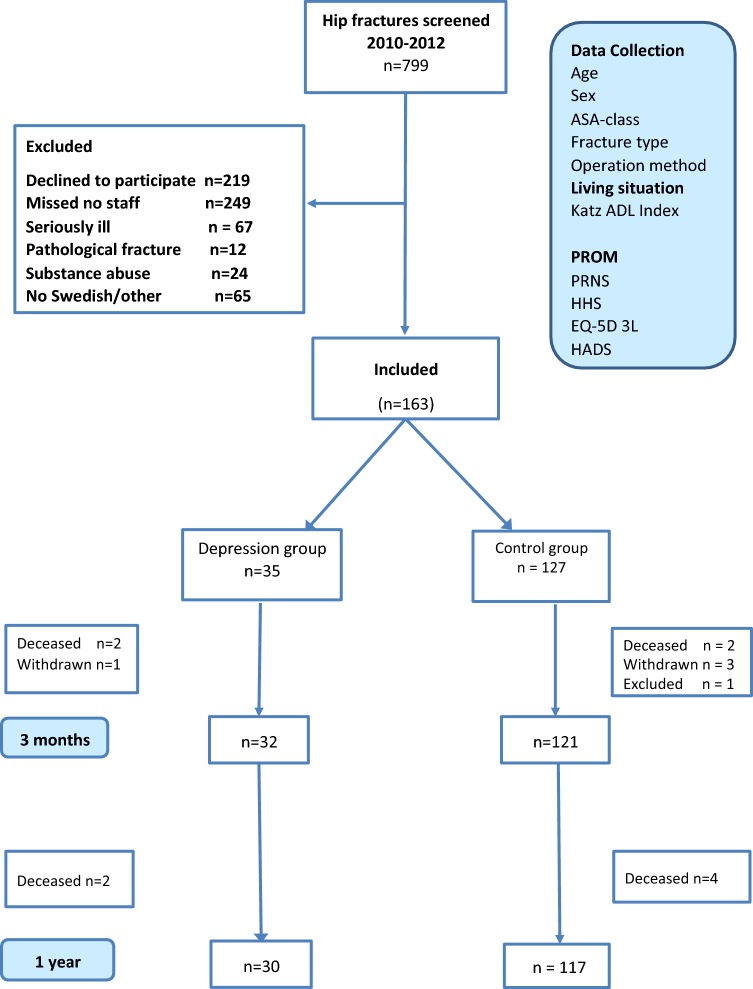
Flowchart showing the progress of the study

Initially, 163 patients were included in the study but 1 patient in the control group was excluded at surgery as had both a fracture and a joint infection requiring a girdlestone operation. A total of 162 patients were included and the characteristics for the depression group (*n* = 35) and control group (*n* = 127) were similar with no statistically significant differences between the two groups in demographic data at baseline (Fig. 1, Table 1). Four patients withdrew from the study before the 3-month follow-up, one in the depression and three in the control group (Fig. 1).

**Table 1:** Baseline demographics

	Depression group (*n* 35)	Non-depression group (*n* 127)
Age (years)		
(Mean, SD)	75.8 ± 11.1	76.5 ± 11.6
Sex, *n* (%)		
Female	21 (60.0%)	89 (70.1%)
Male	14 (40.0%)	38 (29.9%)
Fracture type		
Cervical	20 (57.1%)	78 (61.4%)
Trochanteric	13 (37.1%)	41 (32.3%)
Subtrochanteric	2 (5.7%)	8 (6.3%)
Operation method		
Internal fixation	7 (20.0%)	35 (27.6%)
Arthroplasty	13 (37.1%)	39 (30.7%)
Sliding screw + plate	3 (8.6%)	39 (30.7%)
Intermedullary nail	12 (34.3%)	37 (29.1%)
Living situation		
Lives alone	18 (51.4%)	53 (41.7%)
With others	17 (48.6%)	74 (58.3%)
Nursing home	0 (0.0%)	0 (0.0%)
Charnley class		
*A* (one hip)	26 (74.3%)	110 (86.6%)
*B* (two hips)	7 (20.0%)	11 (8.7%)
*C* (other joints)	2 (5.7%)	6 (4.7%)
Clinical pathway		
Ambulance	10 (28.6%)	49 (38.6%)
Hip process	25 (71.4%)	78 (61.4%)
KATZ ADL index		
1. Independent	94%	95%

No statistically significant differences between groups

### Outcomes and main results

The HHS scores for the depression group compared to the control were significantly poorer at baseline (85 vs. 91 points; *p* = 0.021). At 3 months, this difference had levelled out (68 vs. 69 points) and although the HHS had improved in both groups at 12 months (74 vs. 78 points), they did not regain their pre-fracture levels (Table 2). There was deterioration in hip function over time in both groups, a 13-point drop in the control group as opposed to an 11-point drop in the depression group from baseline level. The depression group had a poorer function from baseline and this remained consistent throughout the study period.

**Table 2:** Differences in functional outcomes between the two groups during the study period

	Control group Mean ± SD (*n*)	Depression group Mean ± SD (*n*)	Mean difference (95% CI)	*p* value
HHS				
Baseline	91 ± 11 (127)	85 ± 13 (35)	5 (1–9)	**0.021**
At 3 months	69 ± 17 (120)	68 ± 15 (32)	1 (− 6 to 7)	0.852
At 12 months	78 ± 17(117)	74 ± 18(28)	3 (− 4 to 10)	0.391
EQ-5D				
Baseline	0.85 ± 0.23 (127)	0.73 ± 0.26 (35)	− 0.08 (− 0.19 to 0.02)	**0.011**
At 3 months	0.71 ± 0.25 (121)	0.69 ± 0.22 (32)	0.02 (− 0.07 to 0.12)	0.633
At 12 months	0.74 ± 0.26 (118)	0.66 ± 0.24 (30)	0,08 (− 0.02 to 0.19)	0.117
PNRS				
Baseline	0.40 ± 1.6 (127)	0.38 ± 1.3 (35)	− 0.25 (− 0.94 to 0.45)	0.474
At 3 months	2.59 ± 1.93 (120)	1.88 ± 1.79 (32)	0.72 (− 0.03 to 1.46)	0.060
At 12 months	1.71 ± 1.86 (119)	2.04 ± 2.17 (28)	− 0.32 (− 1.12 to 0.48)	0.426
HADS				
Baseline	2 ± 2 (127)	6 ± 3 (35)	− 4.37 (− 5.50 to − 3.25)	**0.000**
Normal (0–7)	120 (100%)	17 (49%)		
Abnormal (8–21)	0 (0%)	18 (51%)		
At 3 months	2 ± 3 (118)	4 ± 0.79 (32)	− 2.75 (− 4.46 to − 1.03)	**0.002**
Normal (0–7)	108 (92%)	22 (69%)		
Abnormal (8–21)	10 (8%)	10 (31%)		
At 12 months	2 ± 2 (114)	6 ± 3 (28)	− 3.40 (− 4.71 to − 2.09)	**0.000**
Normal (0–7)	106 (93%)	19 (68%)		
Abnormal (8–21)	8 (7%)	9 (32%)		

Variables are presented as the mean with standard deviation and the mean difference is presented with 95% confidence intervals. *p* values were derived from the Student’s *T* test

Bold* p*-values are statistically significant

*HHS* Harris hip score, *EQ-5D* European quality of life five dimensions, *PNRS* pain numerical rating scale, *HADS* Hospital Anxiety and Depression Score, depression subscale

A statistically significant difference, in the EQ-5D scores for the groups at baseline, was seen (0.85 vs. 0.73; *p* = 0.011), the control group score was higher. The scores at both 3 and 12 months were somewhat lower than the baseline score, so patients in both groups experienced a decline in their quality of life over the duration of the study.

The PRNS scores were marginally lower in the depression group at baseline and 3 months indicating less pain was experienced pre-fracture in this group but by 12 months the depression group score was higher, although the differences never reached statistical significance. Not surprisingly, there were statistically significant differences between the groups in HADS Depression subscale throughout the study (Table 2).

### Linear regression model

Those factors that were found to influence the functional outcome at 1 year were the pre-fracture HHS and the pre-fracture EQ-5D score (Table 3). To which group the patients belonged to did not affect the 1-year functional outcome. In the crude (unadjusted) model for HHS, we found a statistical significance for pre-fracture HHS score, age, and ASA-classification but once the figures were adjusted only the significance of pre-fracture HHS remained. A similar pattern was seen in the model for the EQ-5D values with only the pre-fracture value remaining significant in the adjusted figures. None of the variables tested in the model reached statistical significance with regard to the PRNS score.

**Table 3:** Linear regression model for functional outcomes at 1 year

Variable	Crude^a^			Adjusted^b^		
	Units	95% CI	*p* value	Units	95% CI	*p* value
Harris Hip Score						
Group	− 3.2	− 10.4–4.1	0.391	− 0.6	− 7.0–5.8	0.851
Pre-fracture score	0.7	0.5–0.9	< 0.001	0.7	0.4–0.9	< 0.001
Age	− 0.3	− 0.6–0.1	0.007	− 0.2	− 0.4–0.0	0.099
Sex	− 2.2	− 8.4–4.1	0.488	− 0.6	− 7.1–3.9	0.566
ASA	− 8.6	–14.0–3.0	0.003	− 0.1	− 4.9–2.9	0.596
EQ-5D index						
Group	− 0.08	− 0.19–0.02	0.117	− 0.02	− 0.11–0.07	0.672
Pre-fracture score	0.52	0.37–0.67	< 0.001	0.47	0.31–0.62	< 0.001
Age	− 0.00	− 0.01 to − 0.00	0.016	− 0.00	0.01–0.00	0.230
Sex	− 0.01	− 0.01 to 0.08	0.888	0.01	− 0.07 to 0.09	0.853
ASA	− 0.16	− 0.24 to − 0.08	< 0.001	− 0.05	− 0.11 to 0.00	0.063
PNRS						
Group	0.3	− 0.5 to 1.1	0.426	0.3	− 0.5 to 1.1	0.500
Pre-fracture score	0.2	− 0.0 to 0.5	0.055	0.2	− 0.0 to 0.5	0.087
Age	− 0.0	− 0.0 to 0.0	0.642	− 0.0	− 0.0 to 0.0	0.654
Sex	0.2	− 0.5 to 0.8	0.649	0.1	− 0.6 to 0.8	0.702
ASA	0.2	− 0.4 to 0.8	0.469	0.2	− 0.5 to 0.9	0.584

All outcomes are also adjusted by their pre-fracture status, e.g. the pre-fracture HHS is used as a co-variate in the model for HHS at 1 year, and the same applies for pre-fracture PRNS and EQ-5D values

^a^Unstandardized Coefficients B

^b^The models are adjusted for group (depression/no depression), age, sex and ASA-classification

### Adverse events

The occurrence of patient-reported AEs and SAEs during the study period was higher in the depression group compared to the control group but did not reach statistical significance. The AE rate for the depression group compared to the control group was 40.0% (14 of 35) vs 33.1% (42 of 127), *p* = 0.445. The pattern was similar for SAEs, with 45.7% (16 of 35) in depression group suffering at least one SAE compared to 33.9% (43 of 127) in the control group, *p* = 0.197.

25 (15.4%) of 162 patients suffered falls during the duration of the study, of these 15 patients sustained fractures: 13 (8.0%) with fragility fractures and 2 with fractures adjacent to their prosthesis.

The 1-year mortality was 6.2% (10 of 162) and was higher in the depression group 11.4% (4 of 35), compared to the control group, 4.7% (6 of 127), chi-square test, but this difference was not statistically significant (*p* = 0.145).

## Discussion

In this prospective observational study of hip-fracture patients, we found no correlation between the presence of depressive symptoms and/or a previous depression at baseline and a poorer functional outcome after 1 year in patients with hip fracture.

Although the results of our study contrast with the findings of studies where depression was found to adversely influence outcome after hip fracture [7–9], they are consistent with other studies where no relationship was found [15–17].

The presence of depressive illness prior to hip fracture may be associated with increased frailty at the time of fracture [35] and may cause a delay in regaining physical function in the short term after hip fracture by reducing involvement in rehabilitation activities [36] but pre-fracture depressive illness may have no long-term effect on physical performance [17].

The only factors that we found had a bearing on functional outcome at 12 months were the baseline HHS and EQ-5D scores. The HHS in the depression group was significantly poorer at baseline, but this difference had disappeared at the 3- and 12-month follow-up.

One factor, which may have influenced our findings, was the relatively low mean age of the participants, 76 years compared to the mean age of hip-fracture patients in Sweden of 82 years [37]. One possible explanation for this is that in the acute setting after a traumatic fracture the frail elderly are less likely to want to participate in activities that have the potential to further complicate their lifestyle.

Another reason that our results differ from others is that we excluded patients with cognitive dysfunction from our study. The rationale for this being that the outcome measures were specifically patient-reported questionnaires and we considered the use of proxy responders might have a negative effect on the quality of the data collected.

Of the 162 patients analyzed, 35 patients (21.6%) had either a score indicating pre-op depressive symptoms or had a previous diagnosis of depression. There was a higher proportion of males in the depression group (40% vs. 29.9%), but it is not possible to draw any conclusions regarding gender differences as the groups were small. Those patients included with depressive symptoms had mild or moderate symptoms (HADS–D score 8–14, none had severe symptoms 15–21). This was surprising as the literature tells us that depression is common among hip-fracture patients. One explanation for this could be that those patients with severe symptoms would be less likely to agree to participate in studies, due to the nature of the illness.

In our study, patients with depression had an increased mortality rate at 1 year but this difference was not statistically significant. Other studies have shown depression is associated with an increased mortality [3, 4]. The lower mortality rate found in our study can depend on the fact that the participants were of a younger age and had a lower ASA-classification than the average hip-fracture patient.

In the literature, the patient follow-up times vary widely, ranging from the short term in days up to discharge to the long term up to 2 years after surgery. Our patients were followed up for 12 months after their fracture, as one can typically expect recovery by 3 months but there is scope for improvement in hip function up to 12 months after surgery [38].

### Strengths

The cohort was meticulously followed up with visits to or telephone interviews with a research RN and apart from those who died, few patients were lost to follow-up during the study. In addition, in this study we have examined depression in hip-fracture patients in relation to functional outcome using a disease-specific assessment instrument HHS. We used patient-reported outcome measure questionnaires making the patient the primary source of information without incorporating any observational bias from investigators.

Although HADS has not been validated specifically for hip fracture, it has been widely used and in a Swedish population sample has been found to be useful in gauging the presence of depression and anxiety symptoms [39, 40]. In addition, a large meta-analysis of 747 articles examining the validity of the instrument showed it to perform well in the assessment of symptom severity for both depression and anxiety states in the general population as well as for patients assessed in medical, psychiatric and primary care settings [41].

### Limitations

No formal power calculation was done for the study and we were, therefore, unable to detect small significant statistical differences, but on the other hand these small differences may not be clinically relevant. In addition, a large group of hip-fracture patients was excluded from the study, those with dementia or a cognitive impairment. Cognitive impairment in patients with hip-fracture is common [42]. However, correctly estimating their pre-fracture status is difficult as it would be done by a relative or care giver. Our results are, therefore, applicable to hip-fracture patients without cognitive dysfunction.

In trauma patients, due to the nature of the condition, it is difficult to obtain pre-fracture information, so there is always the possibility of recall bias occurring. This is difficult to avoid, as it is not possible to obtain this information prior to the event and this method has been frequently used in hip fracture studies [43, 44]. Given the limitations, the results from our relatively small cohort should be interpreted with caution. It is possible that we were unable to detect minor differences in functional outcome in this selected patient group.

## Conclusion

In our study, we found that depression does not have a profound bearing on the outcome for hip fracture patients without cognitive dysfunction 1 year after their fracture. The results of this study imply that depression is not of major importance for the rehabilitation of hip function in the short term in younger elderly hip-fracture patients.
